# The effect of remote ischemic preconditioning on serum creatinine in patients undergoing partial nephrectomy: a study protocol for a randomized controlled trial

**DOI:** 10.1186/s13063-018-2820-3

**Published:** 2018-09-04

**Authors:** Min Hur, Sun-Kyung Park, Jungho Shin, Jung-Yoon Choi, Seokha Yoo, Won Ho Kim, Jin-Tae Kim

**Affiliations:** Department of Anesthesiology and Pain Medicine, Seoul National University Hospital, Seoul National University College of Medicine, 101 Daehak-ro, Jongno-gu, Seoul, 03080 Republic of Korea

**Keywords:** Remote ischemic preconditioning, Partial nephrectomy, Acute kidney injury, Urinary biomarker

## Abstract

**Background:**

Acute kidney injury (AKI) may develop during partial nephrectomy due to ischemic reperfusion injury induced by renal artery clamping or surgical insult. The effect of remote ischemic preconditioning (RIPC) on reducing the renal injury after partial nephrectomy has not been evaluated in terms of urinary biomarkers.

**Methods/design:**

We will conduct a randomized controlled trial enrolling the patients who will undergo partial nephrectomy. In the study group, RIPC which consisted of four 5-min cycles of limb ischemia and reperfusion will be conducted after induction of anesthesia. Postoperative serum creatinine values, the incidence of AKI, and urinary biomarkers, including urinary creatinine, microalbumin, β-2 microglobulin, and *N*-acetyl-beta-D-glucosaminidase, will be compared between groups during the postoperative 2 weeks. Regional oxygen saturation on the skin of the contralateral kidney will be measured to evaluate the association between intraoperative regional oxygen saturation values and renal injury of the operating side.

**Discussion:**

We expect that our trial may demonstrate the effect of RIPC on mitigating the immediate postoperative renal injury and improving patient outcomes after partial nephrectomy. Moreover, our patients will undergo ^99m^Tc-DTPA radionuclide scintigraphy to calculate glomerular filtration rate 6 and 12 months after surgery. This data should show the long-term effect of RIPC.

**Trial registration:**

ClinicalTrials.gov, ID: NCT03273751. Registered on 6 September 2017.

**Electronic supplementary material:**

The online version of this article (10.1186/s13063-018-2820-3) contains supplementary material, which is available to authorized users.

## Background

Remote ischemic preconditioning (RIPC) refers to applying one or more cycles of brief, non-lethal ischemia and reperfusion to a distant organ or tissue, which is known to protect heart and other organs against acute ischemic insult [1–4]. To demonstrate the renal protective effect of RIPC, many clinical trials were published under various surgical settings. According to two recently reported meta- analyses, RIPC is beneficial to prevent the development of acute kidney injury (AKI) in cardiac or vascular procedures [5, 6]. A multicenter randomized trial reported that RIPC could reduce the incidence of postoperative AKI and renal replacement therapy during cardiac surgery [7]. However, according to the Cochrane Review, RIPC did not lead to significant differences in serum creatinine, need for dialysis and incidence of AKI in patients who underwent interventions that may result in ischemic renal injury [8]. Thus, there is still controversy about the protective effect of RIPC on the renal ischemic injury.

Partial nephrectomy has now become a standard treatment for localized renal small cell cancer [9, 10]. Partial nephrectomy can preserve normal renal parenchyma and kidney function. The incidence of postoperative AKI and chronic kidney disease were significantly lower after partial nephrectomy compared to radical nephrectomy [11–13]. However, during partial nephrectomy, the renal vascular pedicle usually needs to be temporarily clamped, leading to ischemia-reperfusion injury (IRI). AKI after partial nephrectomy still occurs frequently due to IRI and the incidence of AKI after partial nephrectomy has been reported to be as high as 39% [14].

Theoretically, we assumed that RIPC, which can prevent IRI, may reduce renal ischemic injury in patients undergoing partial nephrectomy. There are only two randomized controlled trials (RCT) which evaluated the effect of RIPC during partial nephrectomy [15, 16]. Although these studies suggested the renal protective effect of RIPC, one study did not measure biomarkers to detect renal injury [15] and another study measured serum biomarkers of neutrophil gelatinase-associated lipocalin (NGAL) and cystatin C only during the immediate postoperative period [16]. However, as other urinary biomarkers, including urinary creatinine, microalbumin, β-2 microglobulin, and *N*-acetyl-beta-D-glucosaminidase (NAG) are also available [17–22], the effect of RIPC can be evaluated with these biomarkers. Glomerular filtration rate (GFR) and split function of each kidney can be measured by technetium diethylene triamine pentacetic acid (^99m^Tc-DTPA) radionuclide scintigraphy. Since AKI can predispose to chronic kidney disease [23], the long-term effect of RIPC in terms of GFR measured by scintigraphy need to be evaluated.

Therefore, we hypothesized that RIPC may mitigate the IRI due to renal arterial clamping during partial nephrectomy, thereby, reducing the elevation in serum creatinine and urinary biomarkers of renal injury. We aimed to evaluate the effect of RIPC on postoperative renal function measured by serum creatinine, urinary biomarkers, and GFR measured by scintigraphy.

## Methods/design

**Fig. 1:**
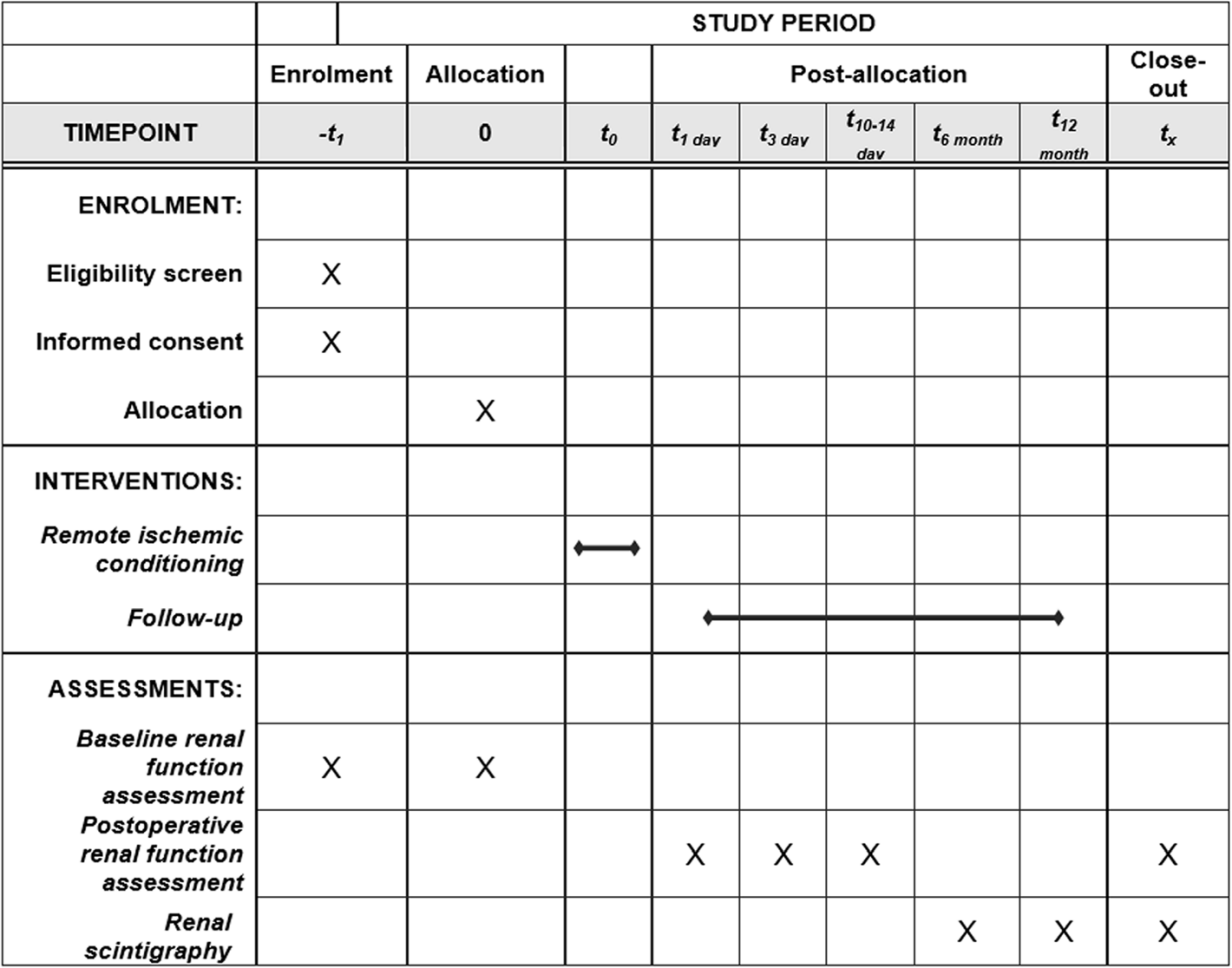
Standard Protocol Items: Recommendations for Interventional Trials (SPIRIT) Figure for the present study

The trial protocol was developed in accordance with the Standard Protocol Items: Recommendations for Interventional Trials (SPIRIT) guidelines [24] (Additional file 1). The SPIRIT Figure on the trial proceedings is added as Fig. 1. This trial is an ongoing, prospective, single-center, surgeon- and patient-blinded, randomized controlled trial.

### Setting and participants

Eligible participants are adults (≥ 20 years of age) who are scheduled to undergo open or laparoscopic or robot-assisted partial nephrectomy. We included patients with a normal contralateral renal function that will be confirmed as a preoperative split renal function of > 40% by the ^99m^Tc-DTPA radionuclide scintigraphy to compare the function of the kidney on the surgery.

Exclusion criteria are the patients with any of the following disease: clinically significant peripheral vascular disease affecting the upper arms, severe cardiopulmonary diseases (valvular or ischemic heart disease, heart failure, left ventricular ejection fraction < 40%, chronic obstructive pulmonary disease, forced expiratory volume in 1 s of < 40% of the predicted value), baseline chronic kidney disease (estimated GFR of < 30 ml per min per 1.73 m^2^ of body-surface area or preoperative serum creatinine level > 1.4 mg/dl), and hepatic failure (bilirubin level of > 1.2 mg/dl or international normalized ratio of > 2.0).

### Informed consent and ethical approval

The subjects to participate in the trial should be informed of all relevant information to this study in terms that they can easily understand. The subjects will be given enough time to consider and have the opportunity to ask questions about the study before deciding to participate in the study. In addition, the subjects should be informed that they will be randomly assigned to the experimental and control groups at a 1:1 ratio, and that they will receive no disadvantage by refusing to participate in the study. After a full explanation, written consent will be obtained from all participants by one of our co-authors. It is a right of the subject to refuse to participate, or to withdraw at any time, during the research period.

This study is being conducted in accordance with the Declaration of Helsinki 1964 as revised in 2013 and the International Conference of Harmonization Guidelines for Good Clinical Practice. This study was approved by the Institutional Review Board of Seoul National University Hospital (approval number: 1707–087-870, protocol version 2.1) and was registered at http://www.clinicaltrials.gov (NCT03273751). We will conduct this study at Seoul National University Hospital. A Data Monitoring Committee was established by the personnel who are independent of study organizers. This committee will monitor participant enrollment, data management, the accuracy of information on the case report form, and compliance to the trial protocol every 6 months.

### Randomization and blinding

After the initial enrollment, participants will be randomly assigned in a 1:1 ratio to either the RIPC or the control group according to Internet-based computer-generated random numbers in block sizes of 4 or 6 (http://www.sealedenvelope.com). Group allocations will be concealed from the investigators using opaque envelopes. The random assignment will be conducted by a third party independent of the study, and the assignment records will not be disclosed until the end of the study. On the day of the surgery, the opaque envelope containing the group allocation will be delivered to an anesthesiologist who is not involved in the study and is responsible for patient care and the RIPC intervention. Participants, urologic surgeons, post-anesthesia care staff, data collectors, and investigators assessing outcome data will be blinded to the treatment allocation to minimize potential sources of bias.

### Surgical and anesthetic procedures

The surgical procedure and anesthesia will be performed according to the standard of our hospital. Anesthesia will be induced with 1.5–2 mg/kg of an intravenous bolus of propofol and continuous remifentanil infusion (effect site concentration of 2–5 ng/ml) using a target-controlled infusion pump (Orchestra®; Fresenius Kabi, Bad Homburg, Germany). After loss of consciousness, rocuronium 0.6 mg/kg will be administered intravenously to facilitate endotracheal intubation. Anesthesia will be maintained with desflurane or sevoflurane using a concentration of 1–1.5 minimum alveolar concentration.

After the patients are put in a right or left upper lateral position for the surgery, a disposable NIRS sensor (INVOS™ Cerebral/Somatic Oximetry Adult Sensor, Medtronic, MN, USA) will be applied directly to the flank area that overlies the opposite kidney that is not undergoing surgery to monitor renal regional oxygen saturation (rSO_2_) under ultrasound guidance [25]. Renal rSO_2_ will be continuously monitored with NIRS (INVOS™ 5100C Cerebral/Somatic Oximeter, Medtronic, MN, USA) until the end of the surgery and rSO_2_ values will be recorded at intervals of 10 min.

### Interventions

After the induction of anesthesia and before renal artery cross-clamping, patients assigned to the RIPC group will receive the RIPC protocol using the upper arm. The RIPC protocol will be performed using an automated cuff-inflator, which consisted of four cycles of 5-min inflations at a blood pressure cuff to 250 mmHg (or at least to a pressure 50 mmHg higher than the systolic arterial pressure), followed by 5-min deflation of a blood pressure cuff. In patients assigned to the control group, a blood pressure cuff will also placed on the upper arm but without cuff inflation during the study period. The intervention will be performed by an anesthesiologist who is independent of this study.

### Study endpoints

**Fig. 2:**
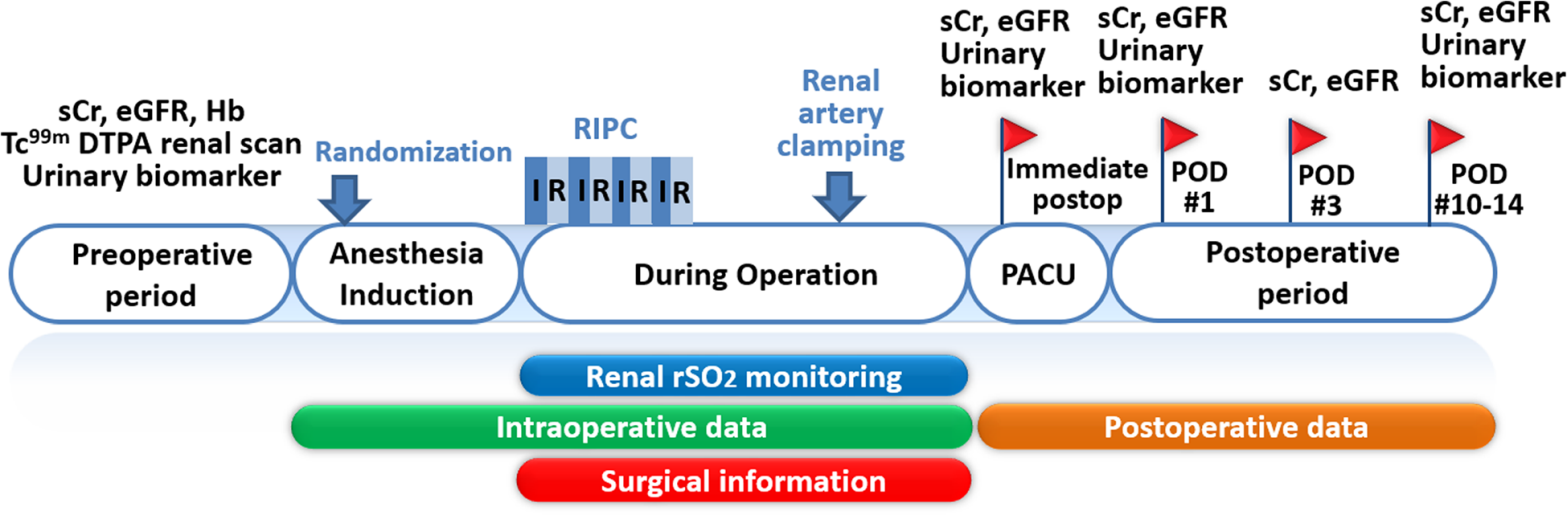
Flow diagram of measurements in the study. *sCr* serum creatinine, *eGFR* estimated glomerular filtration rate, *Hb* hemoglobin, *RIPC* remote ischemic preconditioning, *I*  ischemia, *R*  reperfusion, *rSO*_*2*_ regional oxygen saturation, *PACU* post-anesthesia care unit. Urinary biomarkers, including urinary creatinine, microalbumin, β-2 microglobulin, and *N*-acetyl-beta-D-glucosaminidase will be measured

The primary outcome is serum creatinine level 1 day after partial nephrectomy, as an index of postoperative kidney function. The secondary outcomes include the incidence of postoperative AKI and urinary biomarkers including urinary creatinine, microalbumin, β-2 microglobulin, and NAG measured immediately, and 1 day and 2 weeks after the partial nephrectomy (Fig. 2) [17–22]. The diagnosis of postoperative AKI will be based on the serum creatinine criteria of the Kidney Disease Improving Global Outcomes (KDIGO) criteria [26]. Estimated GFR (eGFR) measured immediately, and at 1 and 3 days, and 2 weeks after partial nephrectomy and renal rSO_2_ of the opposite kidney measured at 10-min intervals from the induction of anesthesia to the end of surgery will also be investigated as secondary outcomes. eGFRs will be calculated with the abbreviated isotope dilution mass spectrometry-Modification in Diet and Renal Disease Study equation, which is $\mathrm{eGFR}\ \left(\mathrm{ml}/\min /1.73\ {\mathrm{m}}^2\right)=175\times {\left(\mathrm{serum}\ \mathrm{creatinine}\right)}^{-1.154}\times {\left(\mathrm{age}\right)}^{-0.203}\times 0.742\ \left(\mathrm{if}\ \mathrm{female}\right)\times 1.212\ \left(\mathrm{if}\ \mathrm{black}\right)$ [27].

Also, GFR measured by ^99m^Tc-DTPA renal scintigraphy will be obtained for a preoperative baseline and at 6 and 12 months after surgery.

### Data collection

Preoperative characteristics including age, sex, Body Mass Index, baseline values of serum creatinine concentration, eGFR, hemoglobin, ^99m^Tc-DTPA renal scintigraphy data, underlying diseases, medication status, smoking, and alcohol consumption will be collected.

We will collect surgery and anesthesia-related data including the R.E.N.A.L. nephrometry score [28], which are summarized in Table 1. Postoperative data will be assessed including the length of hospital stay, length of intensive care unit (ICU) stay, incidences of postoperative complications such as reoperation, postoperative wound infection, venous thromboembolism, myocardial infarction, and cerebrovascular accident.

**Table 1:** Intraoperative data collection

Category	Variables
Surgery-related	Duration of surgery
	Type of partial nephrectomy (open/ laparoscopic/ robotic)
	Cold/warm ischemic
	Duration of renal ischemia
	Estimated blood loss
	R.E.N.A.L. nephrometry score
Anesthesia-related	Mean arterial pressure (lowest and highest)
	Total dose of anesthetics (propofol and remifentanil)
	Mean dose of anesthetics (sevoflurane or desflurane)
	Dose of vasopressors (ephedrine, phenylephrine, and calcium chloride)
	Dose of mannitol infused during surgery
	Urine output
	Total crystalloid/ colloid infused
	Blood transfusion amount
	Intraoperative complications

In our trial, all data will be entered electronically. Participant files are to be stored in numerical order and stored in an accessible and secure manner and place. Participant files will be maintained in storage for 3 years after completion of the study. Electronic data errors will be detected by programs designed to detect missing data or specific errors in the data.

### Safety endpoints

Safety outcomes regarding RIPC will be collected as follows. We will inspect and ask patients about ecchymosis or any skin change and symptoms including paresthesia, pain, or numbness where the RIPC was applied. We will monitor patients with a conventional three-lead electrocardiogram, pulse oximetry, and non-invasive arterial blood pressure measurements. Invasive radial arterial pressure monitoring and arterial blood gas analysis will be performed during surgery.

### Sample size

To determine the sample size, we reviewed serum creatinine changes in 20 patients who underwent partial nephrectomy. The mean value of serum creatinine was 1.60 mg/dl, and the standard deviation was 0.54 mg/dl at 1 day after partial nephrectomy. Assuming that the serum creatinine levels in the RIPC group were significantly lower than the levels in the control group by more than 0.35 mg/dl at 1 day after partial nephrectomy, 39 patients per group were required with a two-sided alpha-error of 0.05 and 80% power. Considering the 10% dropout rate, eight patients were added. The final sample size was determined to be 43 patients per group.

### Statistical analysis

Data will be expressed as mean (standard deviation), median (interquartile range) or the number of patients (%). The normality of distribution of data will be tested by the Kolmogorov-Smirnov test. To compare the outcome variables and baseline characteristics between the RIPC and the control group, Student’s *t* test or the Mann Whitney *U* test will be used for continuous variables depending on the distribution of data and the chi-square test or Fisher’s exact test will be used for categorical variables depending on the expected counts. For the comparison of the time-dependent measurement of serum creatinine, eGFR, urinary biomarkers, and renal rSO_2_ of the opposite kidney, repeated measures analysis of variance will be used to assess the changes within and between groups over time. The presence and incidence of missing data will be reported. Multiple imputations using the Markov chain Monte Carlo algorithm will be performed for handling missing values in our study data. Multivariable logistic regression analysis will be performed to adjust for potential confounding factors known to affect the risk of AKI including surgical parameters shown in Table 1 and patient characteristics (age, sex, history of hypertension, diabetes mellitus, renal insufficiency, and congestive heart failure). We planned to perform subgroup analysis based on surgical modalities and R.E.N.A.L. nephrometry score.

Data will be analyzed using SPSS software (SPSS version 24.0, Chicago IL, USA). G*power (version 3.1.9.2, Universität Düsseldorf, Düsseldorf, Germany) was used to calculate the sample size. A *P* < 0.05 will be considered statistically significant. Bonferroni correction for multiple measurements will be used to reduce type 1 error.

### Dissemination of results

The trial protocol and results will be published in peer-reviewed journals. The results of the trial will be reported in accordance with the Consolidated Standards of Reporting Trials (CONSORT) Statement and its extension to non-pharmacological interventions [29, 30]. Participant-level datasets will be provided to researchers who are willing to perform meta-analysis by contacting the corresponding author.

## Discussion

The present study will test the hypothesis that RIPC performed during partial nephrectomy will reduce the IRI caused by renal artery clamping during surgery as well as the surgical insult. Our primary outcome variable will be serum creatinine value measured on the first postoperative day. However, as serum creatinine may be non-specific and insensitive to detect early subclinical renal injury, urinary biomarkers, as well as other secondary clinical outcomes, will be compared between groups. Our study should be able to give a reliable answer to the question of whether RIPC could protect the kidney from IRI during partial nephrectomy.

Patients who undergo partial nephrectomy are prone to develop postoperative renal parenchymal injury theoretically. Only a few studies reported the incidence of AKI after partial nephrectomy with currently available criteria including RIFLE (risk, injury, failure, loss of function, end-stage renal disease), the Acute Kidney Injury Network (AKIN) or Kidney Disease Improving Global Outcomes (KDIGO). To our knowledge, in two previous studies investigating the development of AKI in patients who underwent partial nephrectomy, the incidences of AKI were reported as 39% and 54% according to AKIN and RIFLE criteria, respectively [14, 31]. Another retrospective study attempting to identify the predictors of 30-day AKI after radical or partial nephrectomy reported that the incidence of AKI, defined as an elevation of serum creatinine > 2 mg/dl above baseline or the need for dialysis, was only 1.8% [32]. This low incidence may be due to the different definition of AKI and different inclusion criteria. Our study will add data about the incidence of AKI defined by KDIGO criteria after partial nephrectomy.

The protective effect of RIPC on vital organs against IRI has been reported in previous animal studies [33–36]. Remote ischemic conditioning provided widespread protection from IRI of the major organs including the liver, kidney, heart, and lung in animal models [34, 37]. As remote ischemic conditioning is a simple, inexpensive, and easily applicable technique in the clinical setting, efforts to translate this beneficial effect of RIPC in the animal studies into a clinical setting have been attempted. One clinical study suggested that remote ischemic preconditioning can prevent contrast medium-induced AKI in patients undergoing elective coronary angiography [38]. Ali et al. reported that RIPC reduced myocardial and renal injury after elective abdominal aortic aneurysm repair [39]. However, a recent multicenter trial with 1612 participants undergoing cardiac surgery reported no benefit of RIPC on the clinical outcomes including cardiac death, non-fatal myocardial infarction, coronary revascularization or stroke [2]. A recent Cochrane systemic review reported that there is no evidence that RIPC has a treatment effect on clinical outcomes. However, RIPC reduced cardiac troponin-T release measured at 72 h after surgery and expressed as area under the curve (AUC) [40]. Also, RIPC reduces the amount of cardiac troponin-I release measured at 48 and 72 h after surgery.

The optimal protocol and regimen of RIPC, including the timing, number of ischemia/reperfusion cycle and duration of each ischemic period, have not yet been established. Important variables in the regimen of RIPC are considered to be the optimal duration of the ischemia, the number of cycles repeated, and the site of application of the ischemia [41].

According to the Cochrane Review of ischemic preconditioning (IPC) for the reduction of renal IRI [8], the most common sites to which the IPC is applied are the upper and lower extremities, and three or four cycles of ischemia and reperfusion are used at 5-min intervals. As the muscle mass is different between upper and lower limb, the choice of the limb may influence the effect of RIPC. To provide enough ischemic insult to the limb to maximize the effect of RIPC, we decided to apply the four cycles of 5 min ischemia and 5 min reperfusion [42].

Regarding the inflation pressure of the cuff, a threshold of 200 mmHg has commonly been used in previous studies to induce ischemia of the upper extremities. However, a 200-mmHg inflation of the blood pressure cuff might be insufficient to occlude arterial blood flow of the upper limb in patients with chronic hypertension [2]. In addition, there were no complications induced by the inflation of cuffs in studies applying RIPC in the upper limb with inflation pressures of 300 mmHg [43, 44] and studies using high inflation pressure reported significant protective effects of RIPC [44, 45].

Recent studies reported that the use of propofol may inhibit the protective effect of RIPC [46–48]. In the two previously reported RCTs which showed no significant effect of RIPC, propofol was used mainly for anesthesia maintenance [2, 49]. Accordingly, in our study, propofol will be used only as an induction agent and anesthesia will be maintained with volatile anesthetics to minimize the effects of the propofol.

To our knowledge, there were two studies that evaluated the effect of RIPC during partial nephrectomy [15, 16]. Huang et al. [15] conducted a randomized trial of 82 patients and reported that the decrease in GFR of the affected kidney at 1 month was significantly less in the RIPC group compared to the control group during laparoscopic partial nephrectomy. However, this study did not evaluate the biomarker of AKI. Recently published, the other study evaluated the effect of late and early RIPC, which was conducted 24 h after surgery and after induction of anesthesia, respectively [16]. Serum NGAL and cystatin C as well as GFR 0, 2, and 6 h after surgery were measured in 65 patients undergoing laparoscopic partial nephrectomy. They reported that serum NGAL and cystatin C were significantly lower in both RIPC groups.

Our protocols have several limitations. First, urinary creatinine, microalbumin, β-2 microglobulin, and NAG were chosen as biomarkers of renal injury. These biomarkers were chosen because they are routinely measured in our clinical practice. However, there are other biomarkers with better performance such as urine tissue inhibitor of metalloproteinase-2 and insulin-like-growth-factor-binding protein 7 [50]. Measuring these biomarkers would enhance the sensitivity to detect and compare the renal injury. Second, although we will perform a randomized trial and adjust for the potential confounders in the multivariable analysis, the use of propofol as an induction agent may suppress the effect of RIPC on the renal dysfunction. Third, the sample size of our study was not calculated to detect a difference in the incidence of AKI, but a difference in serum creatinine level on the first postoperative day. Although a 0.35 mg/dl difference in serum creatinine is greater than a 0.3 mg/dl increase for defining stage 1 AKI in the KDIGO criteria [26], our sample size may not be sufficient to detect any difference in the risk of AKI.

In conclusion, our study protocol will test the effect of RIPC on the serum creatinine values after partial nephrectomy. Furthermore, our study will compare the incidence of AKI among the patients who received RIPC and those who did not. The urinary biomarker levels will help us to discriminate the degree of renal injury between the study groups more precisely. If the result of our trial supports our hypothesis, the next step in our research protocol will be to evaluate the long-term effect of RIPC in this surgical setting. Our patients will undergo ^99m^Tc-DTPA radionuclide scintigraphy to calculate GFR 6 and 12 months after surgery. This data will provide the long-term effect of RIPC in addition to the outcome variables that will be reported according to this study protocol.

## Trial status

The study was initiated in September 2017. By June 2018, 46 patients had been enrolled in this study. Enrollment of 86 patients is expected to be completed in the winter of 2018.

## Additional file

Additional file 1: Standard Protocol Items: Recommendations for Interventional Trials (SPIRIT) Checklist. (DOC 124 kb)

## Abbreviations

^99m^Tc-DTPA: Technetium diethylene triamine pentacetic acid
AKI: Acute kidney injury
AKIN: Acute Kidney Injury Network
eGFR: Estimated glomerular filtration rate
IRI: Ischemia-reperfusion injury
KDIGO: Kidney Disease Improving Global Outcomes
NAG: *N*-acetyl-beta-D-glucosaminidase
NGAL: Neutrophil gelatinase-associated lipocalin
NIRS: Near-infrared spectroscopy
RIPC: Remote ischemic preconditioning
rSO_2_: Regional oxygen saturation
